# Conversations in rectal cancer treatment: a qualitative study of multidisciplinary clinician perspective into non-operative management decision-making discussions

**DOI:** 10.1007/s00520-026-11137-4

**Published:** 2026-08-20

**Authors:** Esra Alagoz, Diana Gutierrez-Meza, Ana De Roo

**Affiliations:** 1 Wisconsin Surgical Outcomes Research Program (WiSOR), Department of Surgery, University of Wisconsin-Madison, Madison, WI USA; 2 Division of Colorectal Surgery, Department of Surgery, University of Wisconsin-Madison, Madison, WI USA

**Keywords:** Rectal cancer, Non-operative management, Shared decision-making, Qualitative, Multidisciplinary

## Abstract

**Purpose:**

For select patients with locally advanced rectal cancer (LARC) who achieve a clinical complete response after neoadjuvant therapy, non-operative management (NOM) offers an alternative to surgery but introduces uncertainty and an intensive surveillance burden. Decisions between NOM and surgery are preference-sensitive and evolve as treatment response becomes clearer. How clinicians operationalize shared decision-making (SDM) for NOM remains poorly understood. We aimed to characterize clinician communication strategies and decision processes surrounding NOM, focusing on risk framing and management of uncertainty.

**Methods:**

Eighteen colorectal surgeons, medical oncologists, and radiation oncologists from two academic medical centers completed 30–60-min semi-structured interviews. Transcripts were coded inductively and analyzed using constant comparison methods and iterative codebook development.

**Results:**

Clinicians described discussions about NOM as a longitudinal, multidisciplinary process that unfolds across multiple encounters and clinical specialties, with medical oncologists and surgeons contributing at different points along the treatment trajectory. Complex information was organized through evidence-based explanations and various risk-framing approaches, allowing patients to engage in iterative deliberation while setting clear expectations about the conditional nature of NOM and the demands of ongoing surveillance. The timing and emphasis of discussions varied by treatment phase and clinical role, which supported gradual patient understanding but also created the potential for unrealistic expectations when recommendations from the treatment team were not clearly aligned.

**Conclusion:**

Decision-making about NOM for LARC unfolds over time under clinical uncertainty and across specialties. Structured, phase-specific communication and improved interprofessional alignment may strengthen shared decision-making in this preference-sensitive context.

## Introduction

Locally advanced rectal cancer (LARC) is traditionally managed with multimodality therapy, including neoadjuvant chemoradiation and chemotherapy followed by total mesorectal excision. While surgical resection remains the standard curative-intent treatment, it is associated with substantial morbidity, including impaired bowel, bladder, and sexual function, as well as the potential need for a permanent stoma. Since initial evidence from two decades ago, subsequent studies have demonstrated that a subset of patients achieving clinical complete response after neoadjuvant therapy may be managed safely with non-operative management (NOM), or “watch-and-wait” [1–5]. NOM can preserve organ function, improve quality of life compared with surgery, and offer comparable oncologic outcomes in carefully selected patients, particularly with rigorous surveillance [3, 6].

Despite increasing recognition of NOM and its inclusion in clinical guidelines, whether decisions are made through shared decision-making (SDM) is unclear. Communication may be inadequate to meet patient needs as evidenced by the differences in patient and clinician attitudes toward NOM [7–9] and increased refusal of recommended surgery, potentially due to miscommunication about its appropriateness [10]. Although patients show interest in NOM [11–13], these discussions are inherently complex and time consuming, complicating the clinical implementation. Clinicians’ experience, specialty, and perceptions of patient preferences and risk tolerance likely influence these conversations.

Given the central role of clinician communication in shared decision-making for LARC, understanding how these conversations occur in practice is critical. In this study, we examined how clinicians across specialties discuss NOM, including how they frame risks, benefits, and uncertainties; describe surveillance and recurrence risk; and support decision-making. By characterizing current practices, we aim to identify opportunities to improve patient-centered counseling for select patients with LARC.

## Methods

### Study setting

Participants were recruited from two large Midwestern academic centers which varied in size, resource availability, and patient populations to capture diverse perspectives. These centers were selected because they had experienced multidisciplinary rectal cancer teams and established protocols for evaluating treatment response and conducting the intensive surveillance required for NOM. The centers varied in size, resource availability, patient populations, and cancer care delivery models. Both centers used multidisciplinary case conferences and generally coordinated specialist visits on the same day and in the same location. At one center, multidisciplinary case conference (“tumor board”) occurred before the visits to establish a preliminary plan; at the other, it occurred afterward to integrate clinician assessments and confirm the plan. Some patients received chemotherapy or radiation at outside institutions, which limited coordination across all treating clinicians. Participants included colorectal surgeons, radiation oncologists, and medical oncologists. Some colorectal surgeons at these centers also performed diagnostic or surveillance endoscopy. We used purposive sampling to recruit clinicians involved in rectal cancer care to enhance diversity in gender, race, and years in practice. Potential participants were identified through administrative rosters and department heads and invited via an introductory email from the Principal Investigator describing the study purpose, eligibility, and participation details. Interested clinicians contacted the research team directly. Reminder emails were sent up to three times and with a final reminder from the principal investigator. Interviews were scheduled at participants’ convenience. Participation was voluntary, oral consent was obtained, and participants were compensated. The study was approved by an Institutional Review Board and deemed exempt due to minimal risk.

### Data collection

Our multidisciplinary team developed a semi-structured interview guide to explore clinicians’ practices and perspectives on NOM in locally advanced rectal cancer. Initial interviews were reviewed to refine question content and flow.

A trained master’s-level qualitative interviewer (DGM) conducted virtual interviews using a HIPAA-compliant video platform. Interviews lasted 30–60 min, were audio-recorded, transcribed, de-identified by trained transcriptionists, and imported into NVivo 12 (Lumivero) for data management.

### Data analysis

Three researchers conducted an inductive thematic analysis [14]. We independently reviewed the first three transcripts to familiarize ourselves with participant accounts [15] and created inductive codes. The team then met to discuss initial codes and through consensus, developed a codebook with code definitions.

After finalizing the codebook, transcripts were divided between two coders (EA, DGM), with a third (ADR) serving as reviewer. Discrepancies were resolved through group discussion during regular meetings to ensure consistency. Memos and annotations were maintained throughout to document analytic insights. Once coding was complete, we created data summary tables for each code to synthesize themes across the three clinician groups. Higher-level analysis was conducted by identifying relationships between codes using constant comparison [16] and mind maps. This manuscript focuses on communication strategies used in NOM decision-making discussions.

## Results

Eighteen clinicians from two academic medical centers were interviewed (Table 1). The mean age was 46.5 years (range 34–58). Most were male (67%) and White, non-Hispanic (83%). The sample included colorectal surgeons (67%), medical oncologists (17%), and radiation oncologists (17%). Experience varied, with 44% in practice fewer than 10 years, 33% for 10–20 years, and 22% for more than 20 years. Ten clinicians (56%) were from Center 1 and eight (44%) from Center 2.

**Table 1:** Clinician characteristics, N=18

Mean age, range (years)	46.5 (34–58)
**Sex**	
Male	12 (67%)
Female	6 (33%)
**Race**	
White, non-Hispanic	15 (83%)
Asian, non-Hispanic	3 (17%)
**Clinician specialty**	
Colorectal surgeons	12 (67%)
Medical oncologists	3 (16.5%)
Radiation oncologists	3 (16.5%)
**Years in practice**	
Less than 10	8 (44%)
10–20	6 (33%)
More than 20	4 (22%)
**Participating center**	
Academic Center 1	10 (56%)
Academic Center 2	8 (44%)
**In 2025, % of practice that is rectal cancer**	
10% or less	2 (11%)
11–25%	12 (67%)
26–50%	4 (22%)
**Year when NOM was incorporated into practice (# of clinicians**)	
2020–2023	10 (56%)
2015–2019	6 (33%)
2010–2014	2 (11%)
**In 2025, % of patients managed with NOM (# of clinicians)**	
10% or less	6 (33%)
11–20%	4 (22%)
21–50%	5 (28%)
50% or more	3 (17%)
**% of patients managed with NOM (# clinicians)**	
Just right	14 (82%)
Not enough	3 (18%)

Clinicians reported that rectal cancer accounted for 11–25% of their 2025 practice volume (67%). Most began incorporating NOM between 2020 and 2023 (56%). Use of NOM varied, with 33% managing 10% or fewer patients with NOM and 17% managing more than 50%. Overall, 82% felt the proportion of patients receiving NOM in their practice was appropriate.

**Fig. 1:**
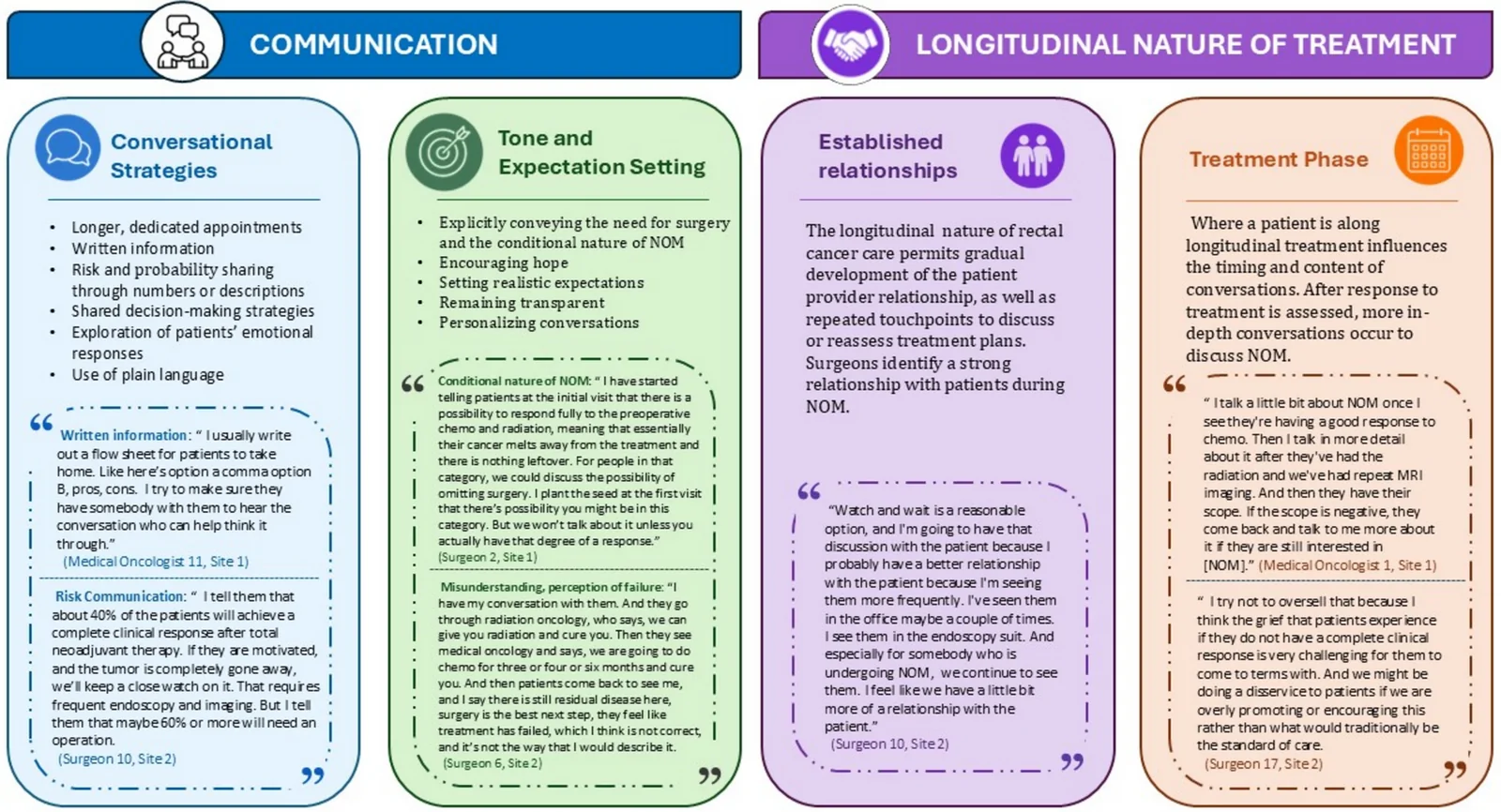
Longitudinal and multidisciplinary communication processes in decision-making about non-operative management

Given this variation in practice patterns, approaches to NOM discussions also differed. Surgeons, medical oncologists, and radiation oncologists approached these discussions from distinct perspectives. Surgeons provided the most detailed descriptions of communication strategies and their use to convey information effectively. Four interrelated themes emerged from the analysis, showing that discussions about NOM unfold as a longitudinal, multidisciplinary process shaped by treatment phase, communication strategies, clinician-patient relationships, and expectation setting (Fig. 1).

### Conversational strategies

Clinicians used various strategies to organize and present complex information about NOM to support patient understanding. Several noted reserving longer or dedicated appointments for these discussions to allow sufficient time for questions and for patients to process the detailed, individualized information.

The treatment of rectal cancer has become extremely personalized. There’s a reason I book hour slots for new patients because we’ve got a lot to think about. And it’s going to vary a lot depending on the particular circumstances of that patient. (Medical Oncologist 1, Site 1)

Many grounded these conversations in evidence, drawing directly from published clinical trial data or institutional outcomes to explain the rationale for recommending or discouraging NOM. Others visually organized information, often by writing out treatment pathways or structured pro–con lists to compare options. Clinicians varied in how they framed risks and probabilities. Some surgeons incorporated numeric estimates when discussing the likelihood of a clinical complete response, local regrowth, or the need for salvage surgery, while others preferred descriptive language rather than percentages, as some patients struggled to contextualize percentages. These numeric or descriptive risk-framing anchors were helpful for grounding discussions and setting realistic expectations.

Clinicians emphasized SDM strategies to support patient deliberation and engagement. Across specialties, they stressed allowing adequate time to consider options and encouraging involvement of family members, second opinions, or independent research, given the complexity and preference-sensitive nature of NOM decisions. To help patients envision treatment pathways, some used anonymized prior patient examples of recovery trajectories, surveillance intensity, and experiences with salvage surgery after local regrowth to make abstract risks more tangible.

If they’re struggling to decide, I’ll say, ‘what additional information would you like? A recommendation from your team? Do you want to see some mannequins that have ostomies on them? Do you want to talk to the ostomy nurse?’ Sometimes, we explore how worried they are about recurrence. That fear of recurrence is really overwhelming and can drive some decision making. (Surgeon 10, Site 2)

Clinicians also described actively exploring patients’ emotional responses, particularly fear of recurrence, as part of these conversations. Finally, across disciplines, participants emphasized the importance of *using plain language*, *minimizing technical or anatomical jargon*, and *tailoring explanations to patients’ health literacy levels*. This was essential for ensuring that patients understood the details of NOM and surgery, felt comfortable asking questions, and could participate meaningfully in shared decision-making.

I try to use layman’s terms and have very informal, open conversations and stay away from heavily scientific terms or anatomical terms when I can. (Surgeon 14, Site 1)

### Tone of conversations and expectation setting

Building on these communication strategies, clinicians described specific approaches to structuring discussion content. For both radiation oncologists and surgeons, a central priority was explicitly conveying the potential need for surgery when considering NOM. They emphasized clearly stating that patients would require surgical resection which potentially involved a permanent colostomy or temporary ileostomy, unless a complete clinical response was achieved and maintained. Establishing this baseline early clarified the conditional nature of NOM and prevented misunderstandings about avoiding surgery altogether.

At the same time, clinicians made a conscious effort to *encourage hope* by presenting treatment options in a positive or optimistic manner, while trying to avoid surgery being seen as failure. Clinicians sought to balance optimism with appropriate caution, noting that excessively positive messaging sometimes caused patients to interpret subsequent surgery as a sign that they had failed treatment. One radiation oncologist emphasized the importance of multidisciplinary alignment, including “making sure that the multidisciplinary team agrees and gives them the same message.” However, clinicians noted that messaging was not always consistent across specialties.

*Setting realistic expectations* was a critical component of these discussions. Clinicians emphasized that NOM requires substantial patient engagement, including adherence to intensive surveillance protocols, frequent imaging, and timely reporting of new symptoms. Many noted that patients often focus on the appeal of avoiding a colostomy bag but may underestimate the long-term commitment and logistical demands of NOM. Ensuring understanding of these requirements was essential for informed decision-making.

It’s more of an informed, mutual understanding of what’s happening for the patient, so a lot of physicians, for someone younger than 30, are a little concerned of watch and wait, given the timeframe that person can recur. But if you have a motivated patient who understands the risks, then I allow them to make their own decision in terms of the opportunities or the alternatives. (Surgeon 3, Site 1)

Across all specialties, the importance of *personalizing conversations* and *remaining transparent* about all available options was highlighted. Discussions were tailored to patients’ knowledge of disease, emotional readiness, and individual goals. Providing a complete, unbiased understanding of both NOM and surgical management was viewed as fundamental to patient-centered care, particularly in LARC, where treatment pathways involve substantial trade-offs and long-term quality-of-life implications.

It’s a very personal decision and there’s a lot of non-black-and-white factors. How anxious are you? What other things are going on in your life? Some patients are, absolutely, I want watch and wait. And some don’t want to do it. The ones in the middle, a heartfelt, thoughtful conversation is going to really help them. (Medical Oncologist 1, Site 1)

### Influence of established relationships

The depth and ease of later discussions were strongly shaped by patient–clinician relationships established earlier in care. Clinicians noted that brief, early introductions to the concept of NOM by multiple team members helped familiarize patients before more detailed discussions. Several also emphasized the advantages of established, longitudinal relationships for decision-making. Surgeons, in particular, were described as having stronger connections with patients due to frequent interactions through preoperative consultations, endoscopy, and follow-up, which fostered trust and rapport. This continuity positioned surgeons as the clinicians patients turned to for detailed explanations and reassurance and facilitated more nuanced discussions of risk, surveillance, and expectations.

### Influence of treatment phase

The phase of treatment strongly shaped both the timing and content of conversations. During the initial consultation, clinicians mentioned NOM only briefly as a potential option, prioritizing establishment of the overall treatment plan and patient understanding of chemoradiation or chemotherapy as the first step. Several clinicians noted that introducing NOM in detail too early could lead to disappointment or grief, complicating later discussions about surgery if a complete clinical response was not achieved. Accordingly, early discussions framed NOM as a possibility rather than a defined pathway.

I feel like some people hear that, this is going to be treated without surgery, and surgery would be a failure. And there must be something wrong with surgery. And then when they finally are told, ‘yes, you need to have surgery’, they dig their heels in. And they’re like, ‘no, I don’t actually want to do anything’. So then, you’re like, ‘maybe I shouldn’t have even offered that in the first place’. (Surgeon 10, Site 2)

Most clinicians deferred detailed discussions of NOM until treatment response could be meaningfully assessed. Following neoadjuvant treatment, when tumor response was clearer, surgeons revisited NOM at restaging; if a complete clinical response was observed, they presented it as a viable alternative to immediate surgery, focusing on surveillance intensity, recurrence risk, and the possibility of salvage surgery. Medical oncologists similarly aligned discussions with the chemotherapy trajectory, introducing NOM after the first 2 to 3 months and revisiting it as patients progressed through treatment phases. This timing allowed oncologists to assess evolving patient priorities, provide education, and prepare patients for more detailed discussions later in care. Surgeons, by contrast, often reserved detailed discussion of NOM until after neoadjuvant therapy, when restaging results indicated whether intensive surveillance was an option. Overall, clinicians agreed that aligning the depth of discussion with the treatment phase improved patient understanding and supported more grounded decision-making as clinical status evolved.

## Discussion

NOM is a feasible alternative to surgery for select patients with a clinical complete response after neoadjuvant therapy, with acceptable oncologic outcomes and noted quality-of-life benefits over surgical management, but with greater uncertainty and surveillance burden [1, 6, 13, 17]. Therefore, the choice between surgery and NOM, when appropriate, is highly dependent upon patient preferences and goals, which requires thoughtful and thorough communication between patients and clinicians.

This study extends the shared decision-making literature by demonstrating that decisions about NOM in LARC unfold as a longitudinal and distributed process rather than a discrete event. Rather than occurring in a single consultation, discussions about NOM were introduced, revisited, and refined across multiple encounters and clinical disciplines, with medical oncologists and surgeons contributing at different phases of the treatment trajectory. This structure enabled the incremental provision of information, supported the development of trust, and allowed clinicians to align recommendations with patients’ evolving understanding, preferences, and tolerance for risk as treatment response became clearer. By situating SDM within the realities of multimodality cancer care and clinical uncertainty, our findings highlight how trust-building, timing, and interdisciplinary coordination function as core mechanisms through which shared decision-making becomes feasible for preference-sensitive and high-stakes treatment decisions such as NOM.

One novel finding in our study is the recognition of the emotional complexity surrounding both surgery and NOM, particularly the need to address potential disappointment and to avoid framing surgery as a failure. Clinicians shared that they made clear that NOM was very conditional, and surgery was the default, to avoid misunderstandings in future conversations. This may reflect the growing number of patients who refuse recommended surgery, potentially due to misunderstandings about NOM [10]. While patients in other studies have shared their desire for hopeful messaging [18], a surgeon in our study shared the challenges faced when other subspecialties, through hopeful messaging to patients about the success of neoadjuvant treatment, may in fact undermine future conversations about surgery if tumor response is insufficient for NOM to be an option. Surgeons also emphasized the importance of presenting both options in neutral language. This may be a way surgeons try to prevent or circumvent emotional responses from patients, when instead these big emotions should be addressed head-on. There are two intentional steps that clinicians can take when patients express disappointment, grief, or even just hopefulness: identify and recognize the emotion, and then respond to that emotion before adding any new information [19].

Clinicians described personalizing discussions, but few described how they ensure patients’ informational needs are met. Prior studies show that patients often desire different information than what is provided, want access to materials after discussions [11, 12, 20], and prefer information in multiple, customizable formats [12, 21]. Patients also value take-home resources to share with family and to revisit after consultations [21]. Future resources should be co-designed with patients and caregivers and informed by structured needs assessments, drawing on instruments such as the Cancer Survivors’ Unmet Needs (CaSUN) measure while incorporating NOM-specific needs across active treatment, restaging, and surveillance. Although decision support tools, including decision aids, have been developed, they are not widely used in practice [22]. Earlier studies indicate mismatched information priorities: surgeons emphasize surgical options and bowel function, whereas patients also seek basic disease information, timelines, next steps, and resources such as support groups and financial guidance [12]. Evaluations of patient educational materials suggest they are often general, lack personalization and anticipatory guidance, but are helpful for later reference [21]. Future research should examine how clinicians tailor information to individual patients and how longitudinal communication aligns with evolving informational needs. It should also evaluate how these communication processes can be supported to enhance continuity, comprehension, and patient-centered decision-making throughout treatment trajectory. Key areas include defining patients’ informational needs at initial consultation, subsequent visits, and the decisional visit for NOM to better support SDM throughout the rectal cancer care.

Including all three clinician groups involved in rectal cancer care highlighted the longitudinal, multidisciplinary nature of information sharing in SDM for LARC. Medical oncologists revisited NOM throughout treatment, enabling incremental information uptake, whereas surgeons typically conducted the most detailed discussions after restaging confirmed a complete clinical response. These discipline-specific patterns indicate that SDM about NOM is distributed across clinicians at different points along the treatment trajectory. This staged approach described in literature [23] is common in patient consultations, and promotes ongoing dialogue, creates opportunities for questions [24], and mitigates information overload through follow-up and check-ins [25, 26]. In rectal cancer, this longitudinal, multidisciplinary approach may be both positive and negative: repeated discussion across specialties can enhance understanding of benefits and drawbacks, while inconsistent messaging may lead to unrealistic treatment expectations especially if chemotherapy or radiation was delivered at an outside institution. Structured interprofessional dialogue, including tumor board discussions or asynchronous messaging, may help unify recommendations and reduce psychosocial distress associated with poor communication [27].

Limitations

Our study has some limitations. The perspectives from clinicians at two academic centers may not capture community settings or institutions with differing experience with NOM including practices in community or private-practice settings. The sample size was small, although inclusion of multiple specialties provided breadth of perspectives. Finally, patient perspectives on NOM discussions were not captured; future research should examine how patients interpret and respond to these communication strategies across different treatment trajectories, including sustained NOM, surgery after incomplete response, and salvage surgery following local regrowth. Longitudinal interviews at key treatment and decision points may capture how experiences and informational needs evolve over time. Additionally, future development of patient educational resources should include input from psychosocial oncology professionals to ensure that informational content is paired with appropriate support for fear of recurrence, disappointment, uncertainty, and treatment-related distress.

## Conclusion

Discussions of NOM for LARC are shaped by treatment phase, clinician specialty, and the clinician-patient relationship. Clinicians use a range of strategies to help patients navigate uncertainty, interpret complex information, and engage in SDM over time. As NOM becomes increasingly integrated into care, not just in rectal cancer, but across different cancer diagnoses where treatment decisions involve trade-off, addressing the emotional complexity of treatment decisions, providing information tailored to individual and treatment-phase needs, and ensuring longitudinal multidisciplinary engagement may improve the quality, clarity, and consistency of these conversations.

## Data Availability

The Authors’ data will not be made available to other researchers.
